# RISK and SAFE Signaling Pathway Involvement in Apolipoprotein A-I-Induced Cardioprotection

**DOI:** 10.1371/journal.pone.0107950

**Published:** 2014-09-19

**Authors:** Hussein Kalakech, Pierre Hibert, Delphine Prunier-Mirebeau, Sophie Tamareille, Franck Letournel, Laurent Macchi, Florence Pinet, Alain Furber, Fabrice Prunier

**Affiliations:** 1 L’UNAM Université, Angers, France; 2 Université d’Angers, Laboratoire Cardioprotection, Remodelage et Thrombose, Angers, France; 3 Université d’Angers, INSERM U771, CNRS UMR 6214, CHU Angers, Département de Biochimie et Génétique, Angers, France; 4 CHU Angers, Laboratoire de Neurobiologie-Neuropathologie, UPRES EA3143, Institut de Biologie en Santé - PBH, Angers, France; 5 INSERM, U744, Institut Pasteur de Lille, Université Lille Nord de France, Lille, France; 6 CHU Angers, Service de Cardiologie, Angers, France; I2MC INSERM UMR U1048, France

## Abstract

Recent findings indicate that apolipoprotein A-I (ApoA-I) may be a protective humoral mediator involved in remote ischemic preconditioning (RIPC). This study sought to determine if ApoA-I mediates its protective effects *via* the RISK and SAFE signaling pathways implicated in RIPC. Wistar rats were allocated to one of the following groups. Control: rats were subjected to myocardial ischemia/reperfusion (I/R) without any further intervention; RIPC: four cycles of limb I/R were applied prior to myocardial ischemia; ApoA-I: 10 mg/Kg of ApoA-I were intravenously injected prior to myocardial ischemia; ApoA-I + inhibitor: pharmacological inhibitors of RISK/SAFE pro-survival kinase (Akt, ERK1/2 and STAT-3) were administered prior to ApoA-I injection. Infarct size was significantly reduced in the RIPC group compared to Control. Similarly, ApoA-I injection efficiently protected the heart, recapitulating RIPC-induced cardioprotection. The ApoA-I protective effect was associated with Akt and GSK-3β phosphorylation and substantially inhibited by pretreatment with Akt and ERK1/2 inhibitors. Pretreatment with ApoA-I in a rat model of I/R recapitulates RIPC-induced cardioprotection and shares some similar molecular mechanisms with those of RIPC-involved protection of the heart.

## Introduction

Although early myocardial reperfusion remains the most effective strategy for limiting myocardial infarct size (IS), it is now well recognized that reperfusion itself can cause harmful myocardial damage [1], [2]. Remote ischemic preconditioning (RIPC) is a powerful phenomenon whereby transient, non injurious ischemia/reperfusion (I/R) episodes are applied to an organ located far from the heart with the aim of protecting the myocardium from I/R injury [3]–[5]. Short I/R bursts applied to various tissues, such as intestine, kidney, liver, or skeletal muscle, have been reported to robustly decrease myocardial injury in different animal models [6], [7] [8]. In an interesting development, RIPC has also emerged as an attractive strategy in a variety of clinical settings [9], [10]. Reported advantages include both reducing myocardial injury and improving outcome in patients undergoing cardiac surgery for congenital heart disease [11] or coronary bypass [12], [13], as well as in those undergoing elective or primary percutaneous coronary intervention [14]–[16]. Nevertheless, our understanding of the underlying mechanisms behind RIPC’s protective effects is still incomplete and the subject of intense research. Current evidence suggests that unknown humoral mediators are released by the remote organ into the circulation, consequently exerting their protective effect on the heart by activating an intrinsic pro-survival signaling cascade [17], [18]. RIPC-induced cardioprotection has been reported to involve two major pathways: 1) the reperfusion injury salvage kinase (RISK) pathway, including phosphatidylinositol 3-kinase/Akt (PI3 K/Akt), extracellular signal-regulated kinase (ERK1/2), and the downstream target glycogen synthase kinase-3 beta (GSK-3β); 2) the survivor activating factor enhancement (SAFE) pathway, including the tumor necrosis factor alpha (TNF-α) and the transcription factor signal transducer and activator of transcription-3 (STAT-3) [19]–[21].

Recent data, obtained from proteomic approaches in rats [22] and humans [23], has indicated that apolipoprotein A-I (ApoA-I) may constitute a protective blood-borne factor involved in the RIPC mechanism. ApoA-I is the major protein component of high-density lipoproteins (HDL). HDL protective effect was first attributed to their ability to promote reverse cholesterol transport that leads to lipids unloading of the atherosclerotic plaque [24], [25]. Increasing evidence suggests that HDL also produce a direct cardioprotective effect in the setting of acute myocardial I/R injury, independent of their atheroprotective effect [26]–[29] and that HDL are also capable of influencing a number of intracellular signaling pathways, in such a way as to protect the myocardium against I/R injury [30]–[33]. While RIPC has been seen to activate RISK and SAFE pathways in animal models, it is not yet clear if ApoA-I-induced cardioprotection shares the same signaling pathways. This study thus sought to investigate whether injecting ApoA-I into a rat model of I/R would mediate its protective effect by activating the RISK and SAFE pathways.

## Materials and Methods

### Ethics statement

All experiments were performed in accordance with the *Guide for the Care and Use of Laboratory Animals* published by the US National Institute of Health [NIH publication 85 (23), revised in 1996]. Protocols have been approved by our regional ethic committee: Comité Régional d’Ethique pour l’Expérimentation Animale-Pays de la Loire (CEEA.2012.50). All surgery was performed under sodium pentobarbital anesthesia, and all efforts were made to minimize suffering.

### Surgical preparation

For all experiments, male adult Wistar rats, weighing 200–250 g, were used. The rats were anesthetized by an intraperitoneal injection of pentobarbital (60 mg/Kg, Ceva Santé Animale, France), orotracheally intubated, and mechanically ventilated with room air by means of a small animal ventilator (SAR-830 A/P, CWE). Body core temperature was continuously monitored throughout the surgical procedure and maintained at approximately 37°C by means of a heating pad connected to a temperature control unit (HB101/2 RS, Bioseb, France). Median sternotomy was performed, and hearts were exposed by removing the pericardium. A 7-0 monofilament suture (Premio 7.0, Peters Surgical) was placed around the left coronary artery and passed through a short piece of tubing in order to create a reversible snare. Coronary occlusion was initiated by clamping the snare onto the epicardial surface directly above the coronary artery. Ischemia was confirmed by the appearance of epicardial cyanosis and dyskinesis of the ischemic region. Reperfusion was achieved following 40 min of occlusion by loosening the snare and confirmed by observing an epicardial hyperemic response.

### Study groups and experimental protocol

**Figure 1:**
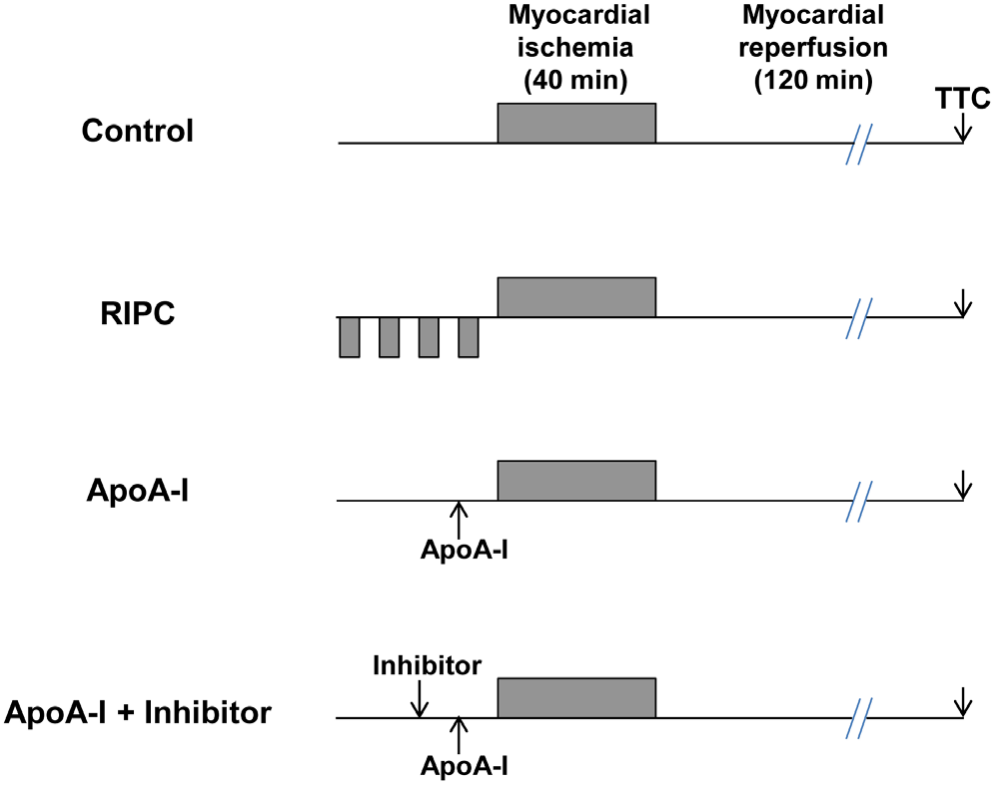
Experimental protocol. In the Control group, rats were subjected to 40 min coronary occlusion followed by 120 min reperfusion without any further intervention. The RIPC group underwent four cycles of RIPC immediately prior to coronary occlusion. ApoA-I (10 mg/Kg) was administered 10 min before coronary artery occlusion (ApoA-I group). Pharmacological inhibitors (either Wortmannin, U0126, or AG490) were administered 10 min prior to ApoA-I injection (ApoA-I + Inhibitor groups). At the end of reperfusion, hearts were excised for infarct size assessment *via* TTC staining.

For the myocardial IS analysis, rats underwent 40 min coronary occlusion followed by 2 hours of reperfusion. They were randomly assigned to one of the following groups (**Figure 1**): 1) Control group (n = 7), with myocardial ischemia (MI) without any further intervention; 2) RIPC group (n = 6), applying RIPC in a sequence of four cycles of 5 min of limb ischemia interspersed with 5 min of limb reperfusion immediately prior to coronary artery occlusion. RIPC was achieved using a vascular clamp placed on the upper right femoral artery in order to occlude arterial blood flow; 3) ApoA-I group (n = 8), applying an intravenous bolus injection of 10 mg/Kg ApoA-I (Academy Bio-Medical Company, Inc. Houston, TX, USA) 10 min prior to coronary artery occlusion. The dose was chosen to assess ApoA-I-afforded cardioprotection in accordance with previous study data describing the protective effects of ApoA-I in doses ranging from 5 mg/Kg to 25 mg/Kg [27], [29], [34]; 4) ApoA-I +wortmannin (Wort) group (n = 7), applying an intravenous bolus injection of Wortmannin (Sigma-Aldrich) (15 µg/Kg), a PI3 K/Akt pathway inhibitor, 10 min prior to ApoA-I administration; 5) ApoA-I +U0126 group (n = 8), applying an intravenous bolus injection of U0126 (Sigma-Aldrich) (200 µg/Kg), an MEK1/2-ERK1/2 pathway inhibitor, 10 min prior to ApoA-I administration; 6) ApoA-I +AG490 group (n = 7), applying an intravenous bolus injection of AG490 (Tocris) (3 mg/kg), a JAK/STAT pathway inhibitor, 10 min prior to ApoA-I administration. At the end of the 2-hour myocardial reperfusion period, rats were euthanized for IS assessment.

For protein phosphorylation state determination, two additional rat groups of six and seven rats respectively, underwent the same experimental procedures as in the Control and ApoA-I groups. Rats underwent 40 min MI and 15 min reperfusion. After 15 min reperfusion, rats were euthanized, their hearts excised, and ischemia-exposed left ventricular tissues collected then freeze-clamped and stored at −80°C for western blot analysis.

For inflammation assessment, two additional rat groups, of three rats per group, underwent the same experimental procedures as in the Control and ApoA-I groups. Rats underwent 40 min MI and 2 hours of reperfusion. Hearts were removed at the end of the reperfusion period and snap-frozen in freeze-gel (Labonord SAS, Templemars, France) for histological and immunohistochemical analysis.

### Area at risk and infarct size determination

At the end of the 2-hour reperfusion period, the left coronary artery was reoccluded using the monofilament suture kept in place and 1% Evans blue was injected for the purposes of delineating the area at risk (AAR). The heart was then cut into five equal slices and incubated with 1% triphenyltetrazolium chloride (TTC, Sigma-Aldrich) pH 7.4 at 37°C for 15 min. The infarcted myocardium within the AAR was not stained and became pale, while the viable was stained deep red. Following overnight storage in 10% formalin, the slices were photographed. IS and AAR were evaluated blindly using Image J software (National Institutes of Health, Bethesda, MD). IS was expressed as a percentage of AAR, and AAR as a percentage of total ventricular area (AAR%LV).

### Western blots

Frozen tissue samples were homogenized in ice-cold lysis buffer consisting of 30 mM HEPES, 20 mM KCl, 2.5 mM EGTA, 2.5 mM EDTA, 40 mM sodium fluoride, 4 mM sodium pyrophosphate, 1 mM sodium orthovanadate, 10% glycerol, 1% Nonidet P-40, phosphatase inhibitor cocktail, and protease inhibitor cocktail. The samples were centrifuged at 13,000 rpm at 4°C for 1 hour in order to isolate total soluble proteins and the resulting supernatant was collected. Protein concentration was determined using the Bio-Rad DC protein assay kit (Bio-Rad, Hercules, CA, USA). Proteins (40–50 µg) were submitted to electrophoresis on a 4–15% denaturing gel (Bio-Rad) at 200 V for 45 min then transferred onto nitrocellulose membranes using the Trans-Blot Turbo Transfer system (Bio-Rad). Once the non-specific binding sites had been blocked with nonfat dry milk in a washing buffer, consisting of 20 mM Tris-HCl, 137 mM NaCl pH 7.6, and 0.1% Tween-20, the membranes were incubated overnight at 4°C with rabbit antibodies for ^473^Ser-phospho-Akt, total Akt, phospho-ERK1/2, total ERK1/2, ^9^Ser-phospho-GSK-3β, total GSK-3β, ^705^Tyr-phospho-STAT-3, and total STAT-3 (all from Cell Signaling Technology). GAPDH (Sigma-Aldrich) was used as a loading control. Once washed in TBS containing 0.1% Tween, the membranes were incubated with horseradish peroxidase conjugated secondary antibodies for 1 hour. Specific antibody binding was detected using electrochemiluminescence. A quantitative analysis of the band densities was performed using Image J software (National Institutes of Health, Bethesda, MD).

### Histological and immunohistochemical analysis of inflammation

For the purposes of investigating histological architecture and leukocyte infiltration, the hearts were sliced into 7 µm-thick transverse sections using Cryostat CM3050 S (Leica Biosystems, Nussloch, Germany). Mid-ventricular sections were stained with hematoxylin and eosine (H&E) then viewed under a light microscope (Leica DMR microscope, Image J software). Immunohistochemical staining for CD45 was performed in order to confirm leukocyte infiltration in infarcted tissues. Mid-ventricular sections were fixed with 4% paraformaldehyde for 30 min, then washed with phosphate-buffered saline and blocked for 30 min with 5% bovine serum albumin at room temperature. They were then incubated overnight at 4°C in a solution of the primary antibody against CD45 diluted to 1∶100 (F10-89-4, Mouse, Millipore). The slices were submitted for stringent wash and incubated with the secondary antibody (1∶300, goat anti-mouse Alexa-Fluor 488, Invitrogen) for 1 hour at room temperature. The nuclei were counterstained with 4′,6-diamidino-2-phenylindole (DAPI) (1∶300, Sigma-Aldrich) for 10 min at room temperature. The slices were washed and coverslips were applied to slides with Fluoromoun aqueous based mounting medium (Sigma-Aldrich). Immunohistochemistry images were taken by means of a Leica microscope (Leica DMR microscope, Image J software). The number of CD45^+^ leukocytes was semiquantitatively evaluated according to the following procedure. Positivity against the antibody was examined and the number of positive cells per field was counted, using 400x magnification. The specimens were scanned randomly, the same field never chosen more than once, by sequential movement of the mechanical stage. We examined 10 fields per rat in the infarcted tissue. The mean number of positive cells was calculated and expressed as the number of positive cells/mm^2^.

### Statistical analysis

All values were expressed as mean±standard error of the mean (SEM). Statistical analyses were performed using SPSS 17.0 (SPSS Inc, Chicago, IL, USA). After testing their linear distribution, the differences between infarct sizes were evaluated using one-way ANOVA, followed by the *post-hoc* Bonferroni test. For the Western blot data, the Mann–Whitney U test was used. A *p* value<0.05 was considered statistically significant.

## Results

### ApoA-I is as effective as RIPC in reducing infarct size

**Figure 2:**
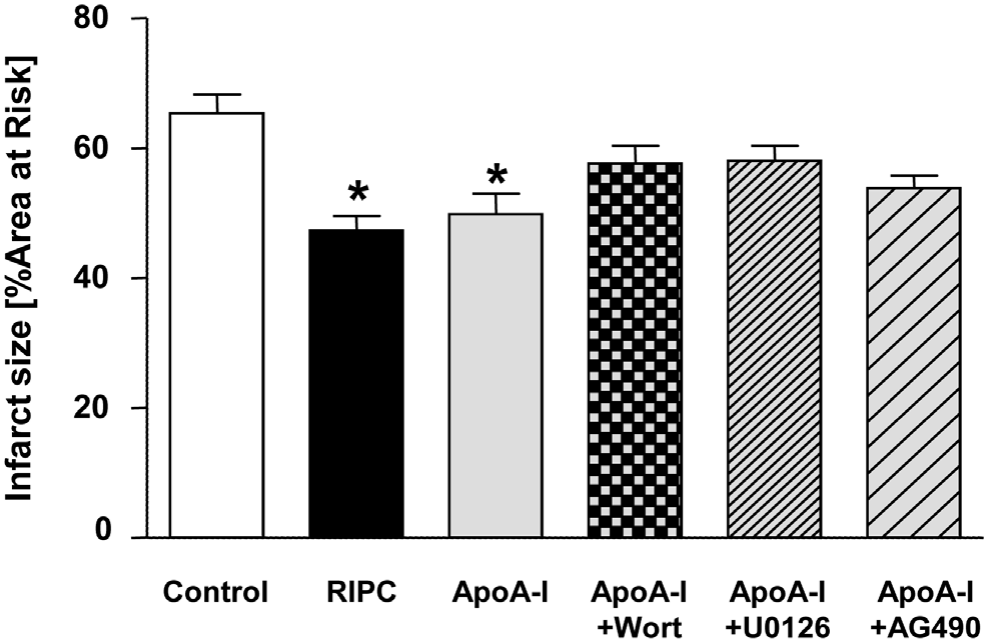
Infarct size expressed as a percentage of area at risk, as determined by TTC staining. Results are expressed as mean±SEM. Wort: Wortmannin, an inhibitor of PI3 K/Akt pathway; U0126: an inhibitor of the MEK1/2-ERK1/2 pathway; AG490: an inhibitor of the JAK/STAT pathway. *p<0.05 *vs.* Control, n = 6–8 in each group.

AAR was comparable among all experimental groups. RIPC induced acute cardioprotection characterized by significant IS decrease (IS/AAR = 65.3±2.9% for Control *vs.* 47.3±2.2% for the RIPC group; *p*<0.001). Similarly, ApoA-I injection significantly decreased IS (IS/AAR = 49.8±3.2%; *p = *0.001 *vs.* Control). IS did not significantly differ between the RIPC and ApoA-I groups (*p = *non significant [ns]) (**Figure 2**).

### Apo-A1 induces the phosphorylation of Akt and the downstream target GSK-3β

**Figure 3:**
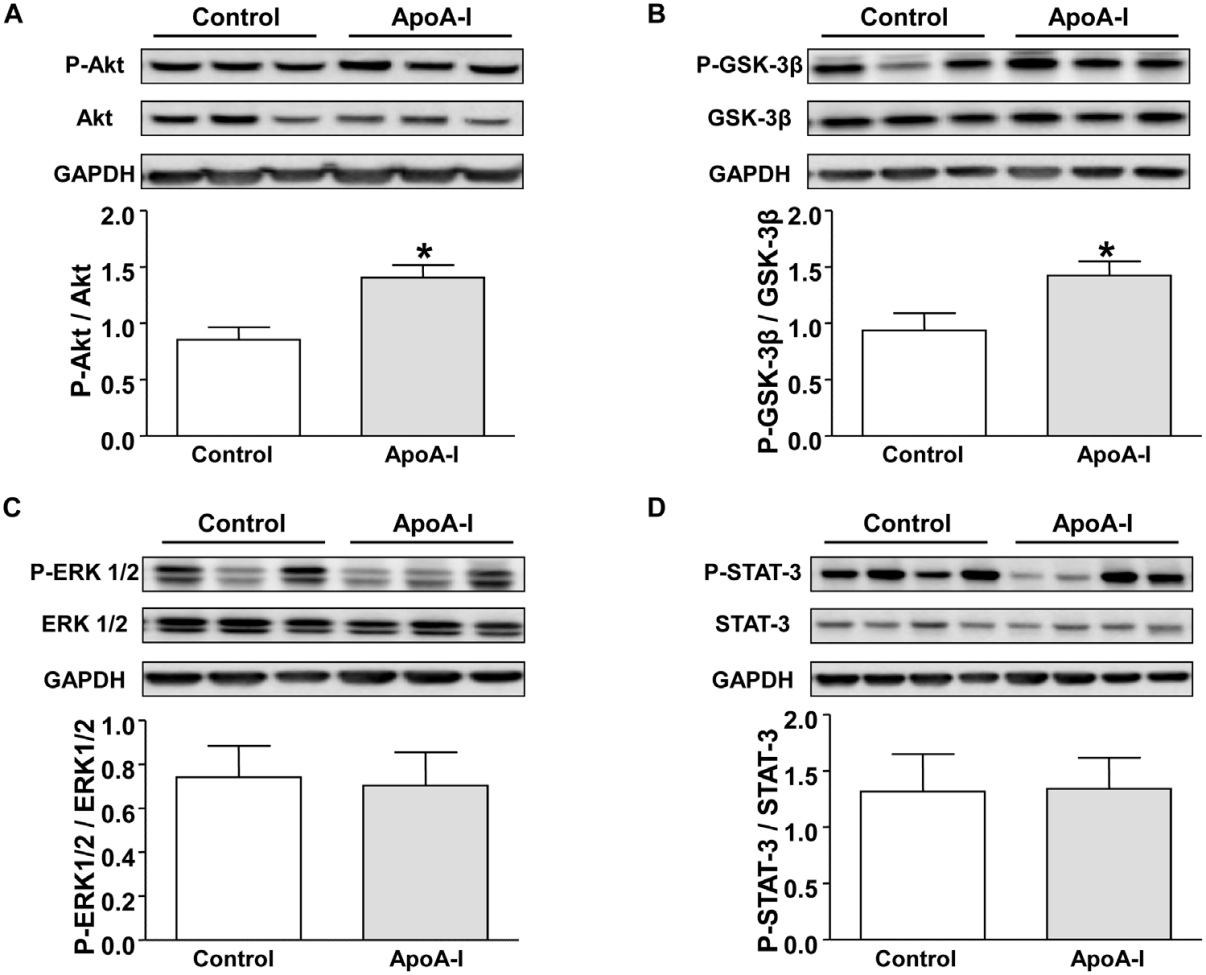
Western blots analysis. Representative immunoblots and densitometric analysis of (A) phosphorylated Akt (p-Akt) and total Akt, (B) phosphorylated GSK-3β (p-GSK-3β) and total GSK-3β, (C) phosphorylated ERK1/2 (p-ERK1/2) and total ERK1/2, and (D) phosphorylated STAT-3 (p-STAT-3) and total STAT-3, at 15 min reperfusion. Results are expressed as mean±SEM. *p<0.05 *vs.* Control, n = 6–7 in each group.

No significant difference was observed in total Akt, ERK1/2, GSK-3β, and STAT-3 protein levels in all groups. We therefore expressed phospho-Akt, phospho-ERK1/2, phospho-GSK-3β, and phospho-STAT-3 levels as densitometric levels normalized by their total protein levels. As illustrated in **Figure 3A**, phospho-Akt levels at 15 min reperfusion were significantly enhanced in the ApoA-I compared to the Control group (*p = *0.005). GSK-3β phosphorylation, a downstream target of Akt, was also significantly increased in the ApoA-I group compared with Control (*p = *0.035) (**Figure 3B**). No significant differences were observed in the phospho-ERK1/2 and phospho-STAT-3 levels between the Control and ApoA-I groups 15 min after reperfusion (**Figure 3C, D**).

### Both the PI3 K/Akt and ERK1/2 pathways are involved in ApoA-I-mediated cardioprotection

In order to further determine the involvement of the RISK/SAFE pathways in ApoA-I-induced cardioprotection, pharmacological inhibitors were administered. As shown in **Figure 2**, the infarct-limiting effect afforded by ApoA-I was inhibited by administering Wort, a PI3 K/Akt signaling pathway inhibitor (IS/AAR = 57.7±2.7%; *p = *ns *vs.* Control). The protective effect of ApoA-I was also inhibited by U0126, the ERK1/2 inhibitor (IS/AAR = 58.8±1.9%; *p = *ns *vs.* Control). AG490, the STAT-3 inhibitor had a modest impact on ApoA-I-induced cardioprotection (IS/AAR = 53.8±1.9%; *p = *0.057 *vs.* Control; *p = *ns *vs.* the ApoA-I group).

### Apo-A1 preserves left ventricular architecture and reduces leukocyte infiltration

**Figure 4:**
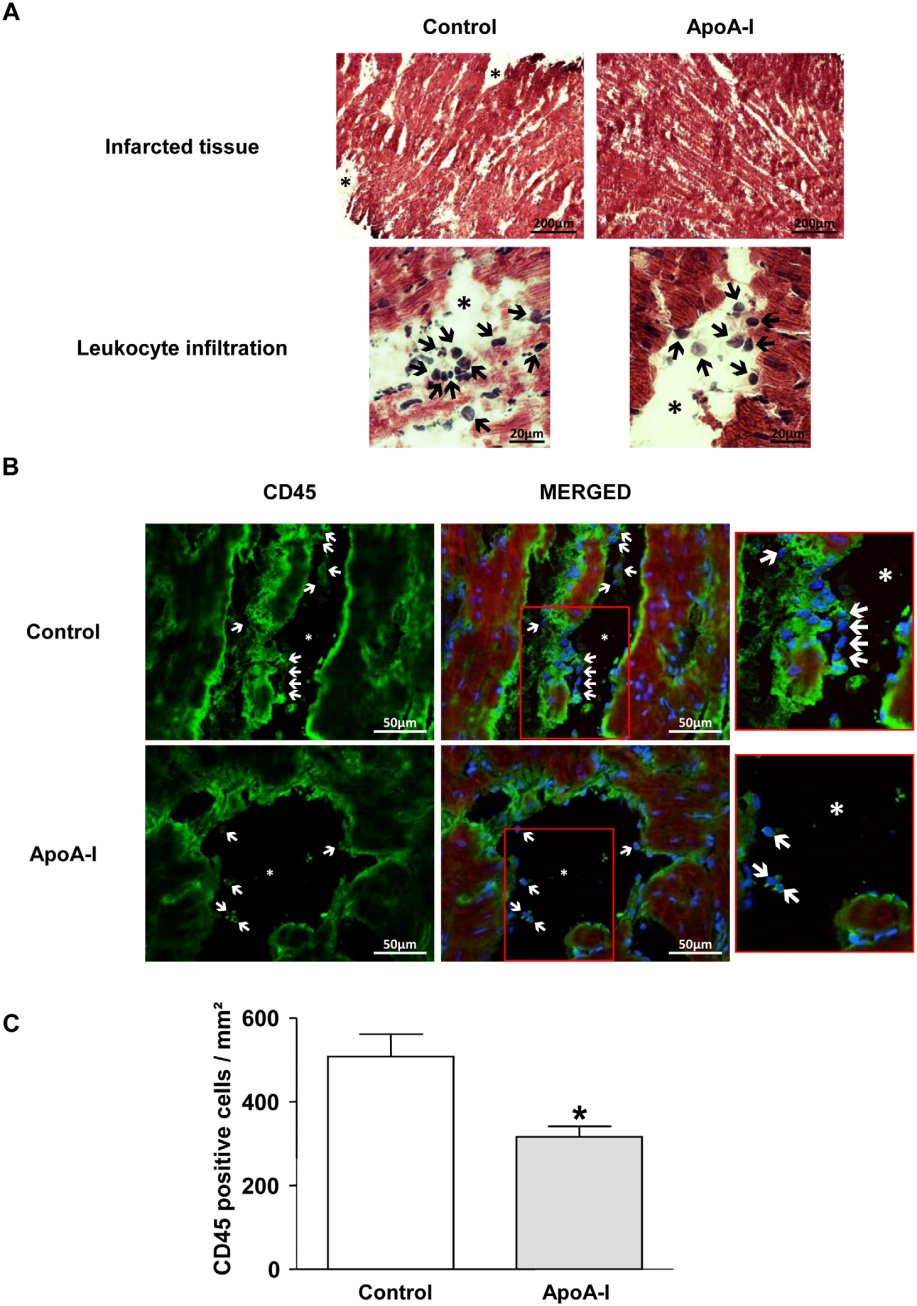
Histological and immunohistochemical analysis of inflammation. (A) Representative photomicrographs of hematoxylin and eosine staining (400x magnification). Ischemic tissue in the Control group revealed frequent myofibril tears and interstitial edema (*) with leukocyte infiltration (black arrows). The ApoA-I group showed reduced myocardial degeneration in infarcted tissue, associated with fewer edema and less leukocyte infiltration. (B) Representative CD45 (green) immunostaining in infarcted tissues (400x magnification). The nuclei were stained with DAPI (blue). Asterisks pinpoint areas of interstitial edema and white arrows indicate some CD45^+^-infiltrated cells. ApoA-I (10 mg/Kg) reduced the infiltration of inflammatory cells in the infarcted region compared to Control. (C) Quantification of CD45^+^ cells in each field. The count was expressed as the number of positive cells/mm^2^. Overall, 10 fields were counted per heart (n = 3 in each group). Results were expressed as mean±SEM. *p<0.05 *vs.* Control group.

In the control rats, hematoxyline-eosine staining revealed signs of extensive damage to infarcted tissues, including myofibril tears, interstitial edema, and leukocyte infiltration. In the hearts from ApoA-I-treated rats, myofibril integrity was better preserved, exhibiting a lower incidence of interstitial edema and less leukocyte infiltration (**Figure 4A**). CD45, also known as leukocyte common antigen, is a transmembrane glycoprotein expressed in significant amounts on the cell surface. When present, it distinguishes leukocytes from non-hematopoietic cells. As was expected, CD45^+^ cells were present in the infarcted tissue of both groups, though the mean number of CD45 positive cells/mm^2^ was limited in the ApoA-I group compared with Control (508±53 cells/mm^2^ in the Control *vs.* 316±25 cells/mm^2^ in the ApoA-I group; *p<*0.05) (**Figure 4B, C**).

## Discussion

Over the last few years, RIPC has emerged as an effective cardioprotective strategy in several experimental studies [7], [20], [35]. The more recent discovery that RIPC stimulus can be induced non-invasively by simply inflating and deflating a blood pressure cuff has encouraged its application in the clinical setting, where it has proven beneficial in a variety of cardiac scenarios [12], [15], [16], [36]–[38]. However, the protective mechanisms by which RIPC exerts its protection are still unclear. A large body of evidence suggests that humoral mediators are released by the remote organ and convey the cardioprotective signal to the heart [17], [18], [39], yet the actual identity of these circulating humoral factors remains unknown. Studies opting for a proteomic approach have suggested that apolipoprotein A-I (ApoA-I), the major protein constituent of HDL, may constitute a protective blood-borne factor involved in RIPC mechanism [22], [23]. Our findings indicate that RIPC and ApoA-I share some common molecular mechanisms by which they protect the heart.

Blood HDL levels are the most powerful independent negative predictor of cardiovascular events in all large prospective epidemiological studies [40]. The long-term benefit of high blood HDL levels has been principally attributed to their key role in reverse cholesterol transport [24], [25]. Short term HDL elevation, however, has also been demonstrated as beneficial [41]. In addition to its reverse cholesterol transport function, HDL also possesses anti-inflammatory, anti-oxidative, anti-apoptotic, and nitric oxide generating properties [42]–[45]. ApoA-I, as a major protein constituent of HDL, is able to recapitulate many of HDL’s protective functions [46], [47]. To date, few studies have been conducted to evaluate the acute cardioprotective effects of ApoA-I/HDL against I/R injury. The protective effect of ApoA-I/HDL in these studies was primarily attributed to their anti-inflammatory and anti-apoptotic properties. HDL potentially reduced IS in mice by inhibiting post-ischemic inflammation and leukocyte recruitment, as well as cardiomyocyte apoptosis [28]. Similarly, ApoA-I administration prior to the onset of reperfusion in rats significantly reduced serum creatine kinase as well as heart TNF-α and interleukin 6 levels, along with reducing endothelial intercellular adhesion molecule-1 (ICAM-1) expression [29]. The mechanisms involved in ApoA-I/HDL-induced cardioprotection have been a subject of particular interest with recent *ex-vivo* and *in-vitro* studies suggesting a direct action of HDL on RISK/SAFE signaling pathways in cardiac cells. In this way, HDL was found to protect cultured cardiomyocytes against hypoxia/reoxygenation damage *via* the activation of the RISK pathway. This cytoprotective effect of HDL was substantially, yet not completely, inhibited by administering pharmacological inhibitors of Akt and ERK1/2 phosphorylation [32]. Furthermore, HDL treatment prevented neonatal cardiomyocytes from doxorubicin-induced apoptosis by activating ERK1/2 and STAT-3. The administration of ERK1/2 or STAT-3 inhibitors reduced the anti-apoptotic effect of HDL, whereas Akt inhibitor had no impact on HDL’s protective effects in this study [48]. Finally, HDL failed to reduce IS in isolated hearts from TNF-α and STAT-3 deficient mice subjected to ischemia [33]. These findings provided evidence for the involvement of the RISK and SAFE signaling pathways in the mechanism of ApoA-I/HDL-induced cardioprotection, as with the mechanisms observed in RIPC [19]–[21]. Nevertheless, there was no information concerning the effect of ApoA-I/HDL on the RISK/SAFE pathways *in vivo*.

In the present study, ApoA-I acutely protected the heart against I/R injury, recapitulating RIPC-induced cardioprotection. This *in-vivo* protective effect generated by ApoA-I can at least partially be attributed to the activation of the RISK/SAFE pathways. ApoA-I injection stimulated the Akt pro-survival signal, which subsequently led to the phosphorylation and hence inactivation of GSK-3β, a known mediator of the inhibition of mitochondrial permeability transition pore opening [19], [20]. In addition, the ApoA-I-induced IS reduction was substantially, though not completely, inhibited by pretreatment with Akt inhibitor. A significant yet not complete inhibition was also achieved with the ERK1/2 inhibitor, although ERK1/2 phosphorylation was not found to be enhanced following ApoA-I injection. This discrepancy may be related to a difference in timing between ERK1/2 activation and tissue samples collection after 15 min reperfusion.

The RISK and SAFE pathways were initially discovered to be protective signaling pathways activated by local ischemic conditioning [49]–[51]. Several studies have previously demonstrated that these pathways also play a role in the cardioprotective effect of RIPC and that the pharmacologic inhibition of these pathways abolishes RIPC-induced cardioprotection [19]–[21]. Tamareille *et al.* have shown that phospho-Akt, phospho-ERK1/2 and phospho-GSK-3β levels were all enhanced by remote conditioning. Furthermore, the administration of Akt and ERK1/2 pharmacological inhibitors abolished remote conditioning protective effects. However, in this study, remote conditioning did not activate phospho-STAT-3 at 15 min reperfusion [21]. Thereby the modest impact of STAT-3 inhibitor on ApoA-I-induced cardioprotection could be understandable. In contrast, Huffman *et al.* have demonstrated that the cardioprotective effects of remote conditioning were mediated by STAT-3 activation and that the inhibition of STAT-3 using AG490 abolished the protection [52]. On the basis of these results, the specific role of STAT-3 in remote cardioprotection still needs to be investigated.

The partial inhibition achieved with pharmacological RISK/SAFE inhibitors in our study could be due to the involvement of other protective ApoA-I functions in terms of acute cardioprotection. Given the now well-documented evidence of ApoA-I’s acute anti-inflammatory properties [45], [53], [54], we therefore elected to assess leukocyte infiltration in the infarcted myocardium following ApoA-I treatment. Histological and immunohistochemical analysis revealed a significant reduction in tissue damage and leukocyte infiltration in ApoA-I-treated hearts. This anti-inflammatory effect of ApoA-I could be the consequence of its role in preventing endothelial dysfunction. In fact, several studies have shown the ability of ApoA-I to reduce the expression of adhesion molecules in endothelial cells and to inhibit the binding of circulating leukocytes to the arterial wall [55], [56]. Interestingly, previous studies have also associated RIPC with anti-inflammatory effects [57]–[59]. Konstantinov *et al.* demonstrated that an RIPC stimulus by brief forearm ischemia suppresses the expression of proinflammatory gene in circulating leukocytes in humans [58]. This alteration in gene expression by RIPC was also accompanied by functional alterations in circulating neutrophils, such as reduced adhesion and phagocytic abilities [57]. Finally, Wei *et al.* have demonstrated that RIPC attenuates neutrophil infiltration in the infarcted myocardium after MI in rats [59].

Taken together, these results indicate that RIPC and ApoA-I share some similar molecular mechanisms by which they protect the heart. Given the fact that plasmatic Apo-A1 levels have been reported to be upregulated following RIPC not only in animals [22] but also in humans [23], RIPC-induced protective effect against I/R injury might thus be partially mediated by ApoA-I.

## Limitation section

This study revealed that ApoA-I and RIPC share similar signaling pathways to protect the heart against I/R injury, yet it did not prove that the cardioprotection induced by RIPC is definitely related to ApoA-I. It would be a fair assumption that ApoA-I does not act solely to mediate the RIPC protective effect. Hepponstall *et al.* and Hibert *et al.* have shown that several humoral mediators are released following RIPC stimulus [23] [22]. These circulating factors may activate several signaling pathways that contribute to the cardioprotective effect.

## Conclusion

While RIPC was shown to activate the RISK and SAFE pro-survival pathways and increase blood ApoA-I levels, our findings indicate that pretreatment with ApoA-I in a rat model of I/R recapitulates RIPC-induced cardioprotection and shares some similar molecular mechanisms by which it protects the heart.
